# Computational identification of antibody-binding epitopes from mimotope datasets

**DOI:** 10.3389/fbinf.2024.1295972

**Published:** 2024-02-23

**Authors:** Rang Li, Sabrina Wilderotter, Madison Stoddard, Debra Van Egeren, Arijit Chakravarty, Diane Joseph-McCarthy

**Affiliations:** ^1^ Department of Biomedical Engineering, Boston University, Boston, MA, United States; ^2^ Fractal Therapeutics Inc., Cambridge, MA, United States; ^3^ Stanford Cancer Institute, Stanford University School of Medicine, Stanford, CA, United States

**Keywords:** conformational epitope, mimotopes, structure-based mimotope mapping, antigen-antibody binding-site prediction, immunodiagnostics design

## Abstract

**Introduction:** A fundamental challenge in computational vaccinology is that most B-cell epitopes are conformational and therefore hard to predict from sequence alone. Another significant challenge is that a great deal of the amino acid sequence of a viral surface protein might not in fact be antigenic. Thus, identifying the regions of a protein that are most promising for vaccine design based on the degree of surface exposure may not lead to a clinically relevant immune response.

**Methods:** Linear peptides selected by phage display experiments that have high affinity to the monoclonal antibody of interest (“mimotopes”) usually have similar physicochemical properties to the antigen epitope corresponding to that antibody. The sequences of these linear peptides can be used to find possible epitopes on the surface of the antigen structure or a homology model of the antigen in the absence of an antigen-antibody complex structure.

**Results and Discussion:** Herein we describe two novel methods for mapping mimotopes to epitopes. The first is a novel algorithm named MimoTree that allows for gaps in the mimotopes and epitopes on the antigen. More specifically, a mimotope may have a gap that does not match to the epitope to allow it to adopt a conformation relevant for binding to an antibody, and residues may similarly be discontinuous in conformational epitopes. MimoTree is a fully automated epitope detection algorithm suitable for the identification of conformational as well as linear epitopes. The second is an ensemble approach, which combines the prediction results from MimoTree and two existing methods.

## 1 Introduction

Antibodies are produced by B cells during the immune response to the presence of foreign matter within the body (“antigens”). Antibodies recognize and bind to specific regions of target antigens to perform their functions, and the regions of antigen molecules to which antibodies attach are defined as epitopes (Sela-Culang et al., 2013). B cell epitopes can be classified into linear and conformational epitopes; in the latter case, epitopes consist of patches of residues that lie close to each other in three-dimensional space but are separated in amino acid sequence (Potocnakova et al., 2016; Sanchez-Trincado et al., 2017). Conformational epitopes generally account for about 90% of overall antibody binding to an antigen (Barlow et al., 1986; Van Regenmortel, 1996). In addition, conformational epitopes may offer functional advantages over linear epitopes that confer enhanced viral neutralization (Vinion-Dubiel et al., 2001) and longer lasting immunity (Steimer et al., 1991). For example, conformational epitope-targeting antibodies to the HIV gp120 glycoprotein are more effective at neutralizing viral isolates than linear epitope-targeting antibodies to the same protein, and they are also responsible for the majority of gp120-specific CD4-blocking activity in HIV-1-infected human sera.

A deeper knowledge of the structure and immunogenic properties of conformational epitopes may be beneficial for designing interventions for many therapeutic areas, including viral infections and cancer (Tegoni et al., 1999; Cho et al., 2003; Adams and Weiner, 2005; Riemer et al., 2005b; Li et al., 2005; Nybakken et al., 2005; Saxena et al., 2006). Immune responses elicited by partial antigens may sometimes be sufficient to provide competent protection since most of the surface structure of the antigen molecules are not antigenic (Sanchez-Trincado et al., 2017). Therefore, identifying immunogenic conformational epitopes and developing strategies to generate an effective immune response against the identified epitopes are important problems in computational vaccinology.

A mimotope is a peptide that mimics the structure of an epitope, and which, in its most strict definition, causes an antibody response that is similar to the one elicited by the epitope (Geysen et al., 1986). An antibody for a given antigen is expected to recognize mimotopes which mimic the corresponding epitope. Mimotopes are commonly obtained from phage display libraries through bio-panning experiments (Bazan et al., 2012). Bacteriophages displaying potential mimotope peptides are incubated with the target antibody which is immobilized on a solid support. As such, specific phages in the library bind to the target antibody. Unbound phages are washed out, while bound phages are selected, eluted, and amplified. Multiple rounds of evolution may be performed. This method is intended to select peptides with high binding affinity to the chosen antibodies. A portion of the selected mimotopes will have some homology to antigen epitopes and may trigger the anticipated immune response. As a result, mimotopes can potentially be employed in and used as a starting point for vaccine design (Bakhshinejad et al., 2016; Goulart and de S. Santos, 2016).

Studies, however, suggest that peptide antigens may not always be capable of generating a sufficient immune response (Chen et al., 2009; Irving et al., 2010; Van Regenmortel, 2016). That is, antipeptide antibodies, raised against peptide antigens, may fail to bind the cognate protein antigens which are in a folded conformational state. Peptides likely exist in an ensemble of conformational states; they may predominantly exist in a linear or disordered state but be capable of adopting distinct conformations a significant percentage of the time. Those peptides that can mimic conformational epitopes found in protein antigens are expected to be able to bind antibodies generated against said protein antigens. Mimotopes, therefore, may be useful in the design of antigens for use in antibody detection in immunodiagnostic tests. Specifically, design of a set of mimotopes that mimic conformational epitopes as the antigens is of high utility in the development of immunodiagnostics to detect antibodies that recognize the corresponding conformational epitopes. Furthermore, mimotopes obtained by panning a phage-displayed random peptide library against a monoclonal antibody or specific sera may produce “target-unrelated peptides” (Huang et al., 2014) in addition to true high affinity binders; computational methods that can map the resulting peptide hits to an antigen structure may, therefore, also be useful for eliminating non-specific peptide binders since those peptides would be less likely to map to the antigen surface.

The algorithmic task of the mimotope-to-epitope mapping is to map the mimotope to the part of the antigen surface that binds the antibody used to create the mimotope. This epitope should have similar physicochemical properties to the mimotope, facilitating antibody binding to both. Since the mimotope is usually not identical but similar to its corresponding epitope at the sequence and structural level, the algorithm considers residues with a physicochemical property distance within a certain range as identical, which is a major challenge in the mapping process. The regions of the antigen surface that are aligned with the mimotopes have a higher probability of being antigenic. Similar to phage-display experiments, molecular docking has been used to enrich potential target regions of the antigen. Protein-protein docking methods have been used to identify the antibody binding site (the epitope) on an antigen protein (Desta et al., 2022); this approach requires a structure or model of both the antigen and antibody structure. Here we are mapping the mimotope to the surface of the antigen to identify the epitopes that the antibody is expected to be capable of binding. As such, only the structure or model of the antigen protein is required. That is, the process of mapping mimotopes (peptides with high binding affinity to the antibody) to the surface of the antigen identifies antibody binding sites on the antigen.

Several computational methods for mimotope mapping based on the antigen structure alone exist (Moreau et al., 2006; Bublil et al., 2007; Mayrose et al., 2007; Negi and Braun, 2009; Chen et al., 2011; Sun et al., 2016), all of which have been validated on a similar set of mimotopes and corresponding protein epitopes based on the existence of the corresponding antigen-antibody complex structures. In general, the overall sensitivity is lower than desired, meaning that residues that lie within the true epitopes are left out of the epitopes predicted by these algorithms. Thus, the existing methods are useful but imperfect.

Specific mimotope-to-epitope mapping algorithms display unique strengths and weaknesses. For example, Mapitope (Bublil et al., 2007) is an algorithm that breaks each mimotope in the dataset into overlapping residue pairs (each residue pair contains two consecutive amino acids in the mimotope sequence), and then computationally pools these residue pairs and ranks the occurrence of each type. Next, it uses the high-frequency occurrences to search on the antigen surface to find the antigen surface residues mapped by these pairs. However, this algorithm requires many statistically relevant parameters customized by the user, and the length of the residue pairs it considers is so short that different parameter settings can have a very large impact on the prediction.

Pupko and co-workers also developed PepSurf (Mayrose et al., 2007), which uses a color-coding algorithm to find all possible simple paths on the antigen surface, matches these paths with mimotopes based on amino acid similarity, and finally clusters the paths with high similarities to get the final prediction. However, the run time of PepSurf depends linearly on the length of the mimotopes because the step of searching for every possible simple path on the antigen surface limits the rate to a great extent. Therefore, PepSurf is only able to process mimotopes with up to 15 amino acids. Subsequently, EpiSearch (Negi and Braun, 2009) solved the run time issue, being able to complete all calculations in less than a minute. However, EpiSearch’s method of dividing regions on the antigen surface is fast but imprecise, and it is also unable to process more than 30 mimotopes at a time.

The overall goal of this study is to develop mimotope-to-epitope mapping prediction methods that are more robust in terms of providing predictions with improved sensitivity. While the mimotope is similar to but usually not identical to the true epitope, most mimotope-to-epitope alignments produce some gaps in mimotope sequence (stretches of the mimotope sequence that do not map to the epitope but rather may simply allow the mimotope to adopt a conformation relevant for antibody binding) when the mimotopes are mapped to the antigen surface. In addition, most contiguous mimotope residues correspond to discontinuous binding sites on the antigen surface that bind to the antibody (Cho et al., 2003). To address this feature, we developed a prediction algorithm that takes into account these sequence gaps while also referring back to the three-dimensional (3D) structure of the antigen in the final mimotope mapping step to achieve more sensitive and accurate epitope prediction. We then explored ensemble approaches combining the new approach with existing methods.

## 2 Methods

### 2.1 Test set

To assess the performance of the mimotope-to-epitope mapping methods, we collated ten test cases from similar publications (Huang et al., 2008). The criteria for selecting test cases are i) the 3D crystallography structure of the antigen-antibody is available; ii) the complex contains only the antigen and antibody; iii) a set of mimotopes was derived from bio-panning experiments with the given antibody. The test set is presented in Table 1, and the sequences for the mimotope sets associated with each test case are given in Supplementary Table S1. Two of the test cases, 1AVZ and 1HX1, are for protein-protein interactions where one protein is considered as the “antigen” here since phage-display libraries of peptides were screened for binding to the other protein considered as the “antibody” in the pair for our purposes. For example, for 1AVZ, a peptide library was screening using phage display to identify peptides that bind to the Fyn SH3 domain (the “antibody”) to block its interactions with negative regulatory factor (Nef) (Rickles et al., 1994; Greenway et al., 1996). The Nef dimer, the biologically relevant form of Nef (Arold et al., 1997; Wu et al., 2018), was taken as the “antigen”.

**TABLE 1:** Mimotope-to-epitope test set.

PDB ID^a^	Antibody	Antigen	References	Library size^b^
Antibody—Antigen Test Cases				
1JRH	mAb A6	IFNgammaR	Lang et al. (2000)	59*5
1BJ1	rhuMAb VEGF	Vascular endothelial growth factor	Chen et al. (1999)	36*6, 3*5, 2*4
1G9M	mAb 17b	Gp120	Enshell-Seijffers et al. (2003)	10*14, 1*12
1E6J	mAb 13b5	P24	Enshell-Seijffers et al. (2003)	14*14, 2*7
1N8Z	Herceptin Fab	Her-2	Riemer et al. (2005a)	5*12
1IQD	mAb Bo2C11	Coagulation factor VIII	Villard et al. (2003)	27*12
1YY9	Cetuximab Fab	Epidermal growth factor receptor	Riemer et al. (2005b)	4*10
2ADF	82D6A3 IgG	Von Willebrand factor	Vanhoorelbeke et al. (2003)	2*15, 3*6
Protein—Protein Test Cases				
1AVZ	Fyn SH3 domain	Nef	Rickles et al. (1994)	8*10, 10*12
1HX1	Bovine Hsc70	Bag chaperone regulator	Takenaka et al. (1995)	8*15

^a^ The protein data bank ID for the antigen-antibody complex X-ray structure.

^b^ Number of sequences (mimotopes in the set) * sequence length.

Since mimotope-to-epitope methods are designed to predict the epitope on an antigen surface, as a more stringent test, we also compiled a set of unbound antigen structures when available that correspond to the test set antigen-antibody structures. This is a more rigorous test since the antigen may undergo conformational changes upon binding to the antibody. The antigen-only test set is given in Table 2. For two of the test cases in Table 1, 1N8Z and 1JRH, the corresponding unbound antigen structure does not exist.

**TABLE 2:** Antigen-only structures corresponding to the test set.

Antigen only PDB ID	Corresponding complex PDB ID	Antigen	References
1VPF	1BJ1	Vascular endothelial growth factor	Muller et al. (1997)
3DNO	1G9M	Gp120	Liu et al. (2008)
1A8O	1E6J	P24	Gamble et al. (1997)
1D7P	1IQD	Coagulation factor VIII	Pratt et al. (1999)
1NQL	1YY9	Epidermal growth factor receptor	Ferguson et al. (2003)
1AO3	2ADF	Von Willebrand factor	Bienkowska et al. (1997)
1AVV	1AVZ	Nef	Arold et al. (1997)
1I6Z	1HX1	Bag chaperone regulator	Briknarová et al. (2001)

### 2.2 Epitope definition

Chimera (Pettersen et al., 2004) was used to define the true epitope for each antigen in the test set. An antigen residue is considered to be part of the epitope if the difference between its solvent-accessible surface area (SASA) in the antigen structure (taken from the complex) and in the antigen-antibody complex is greater than 10 Å^2^ (Hu and Yan, 2009).

### 2.3 Amino acid similarity

Since the amino acids in a mimotope are not typically identical to those in the true epitope, the ability of a mimotope to bind with high affinity to a target antibody and possibly achieve the same function as the true epitope is likely due to the fact that at least a portion of the amino acids in the mimotope share similarity rather than identity with the epitope residues. Similarities between amino acids determine whether they can substitute for each other in a sequence while maintaining similar peptide/protein properties and function. Several methods exist for assessing amino acid similarity, based on the physicochemical properties of the individual amino acids, the role they play in the protein structure, or more subtle contributions (Stephenson and Freeland, 2013). We used quantitative descriptors of the properties of amino acids published by Braun and co-workers when performing mimotope to antigen surface mapping (Venkatarajan and Braun, 2001). The calculated similarity is as follows:

$$PD\left(A,B\right)=\sqrt{\sum_{i}{\lambda_{i}\left(E_{i}\left(A\right)-\;E_{i}\left(B\right)\right)}^{2}}$$

where PD (A, B) represents the property distance between amino acid A and amino acid B, and λ_*i*_ are the eigenvalues corresponding to the eigenvectors E_i_ (i = 1–5) representing the physicochemical properties of each amino acid. The lower the PD between two amino acid types, the more similar they are. According to Braun and co-workers, the finest grouping of amino acids based on the physicochemical properties is defined by a distance cutoff of 9.5 (Venkatarajan and Braun, 2001). In practice, we chose a distance cutoff of 8 based on the run time of the algorithm and accuracy of the output, which means that two amino acids with a PD of 8 or less are considered identical.

### 2.4 Selection of surface residues

In order to perform mimotope mapping only on the antigen surface, we defined antigen surface residues as those having a SASA greater than 5% of their maximal SASA (Miller et al., 1987). The SASA value per residue in the antigen structure was calculated using Chimera with a default probe radius of 1.4 Å (Pettersen et al., 2004). The maximal SASA of each standard amino acid was defined as the total SASA of the residue in an extended Gly-X-Gly peptide where X represents the residue of interest (Miller et al., 1987).

### 2.5 MimoTree

Mimotopes may only partially mimic the structure and function of the true epitopes that they mimic; most mimotopes are similar but not identical to the true epitope both at the level of sequence and structure. Moreover, during the process of the mimotopes binding to the antibody, it is likely that some residues of the mimotopes will not be bound to the antibody residues, but rather will simply exist in the mimotope sequence to allow the mimotope to adopt a conformation relevant for binding to the antibody. As a result, there may be gaps in the mapping of the mimotope sequence to the antigen surface. To address this issue, some existing methods use a gap penalty parameter added to the similarity score of the mimotope and the antigen surface area or to the weighted score of the predicted path, such that the weight of a mimotope that forms gaps is reduced. Introducing a gap penalty, however, reduces the probability that a mimotope with a gap will be highly scored even if it does match a true epitope (Mayrose et al., 2007; Negi and Braun, 2009).

In fact, mimotopes that form gaps during binding should be scored equally well when mapped onto an epitope region. A unique feature of our algorithm is that it allows for these gaps and does not penalize mappings with such gaps. These gaps in the mimotope sequence binding to the antibody are expected to translate into gaps in the mapping of the mimotope onto the antigen surface. Another unique feature of MimoTree is that the 3D structure of the antigen is considered when determining if a gap in the putative epitope mapping on the antigen surface is allowed. That is, if the distance on the antigen surface is within the linear length possible for the number of residues of the mimotope gap, then the mimotope can be mapped at the corresponding position on the surface antigen. The MimoTree code is available on GitHub.

#### 2.5.1 Algorithm flow

**FIGURE 1:**
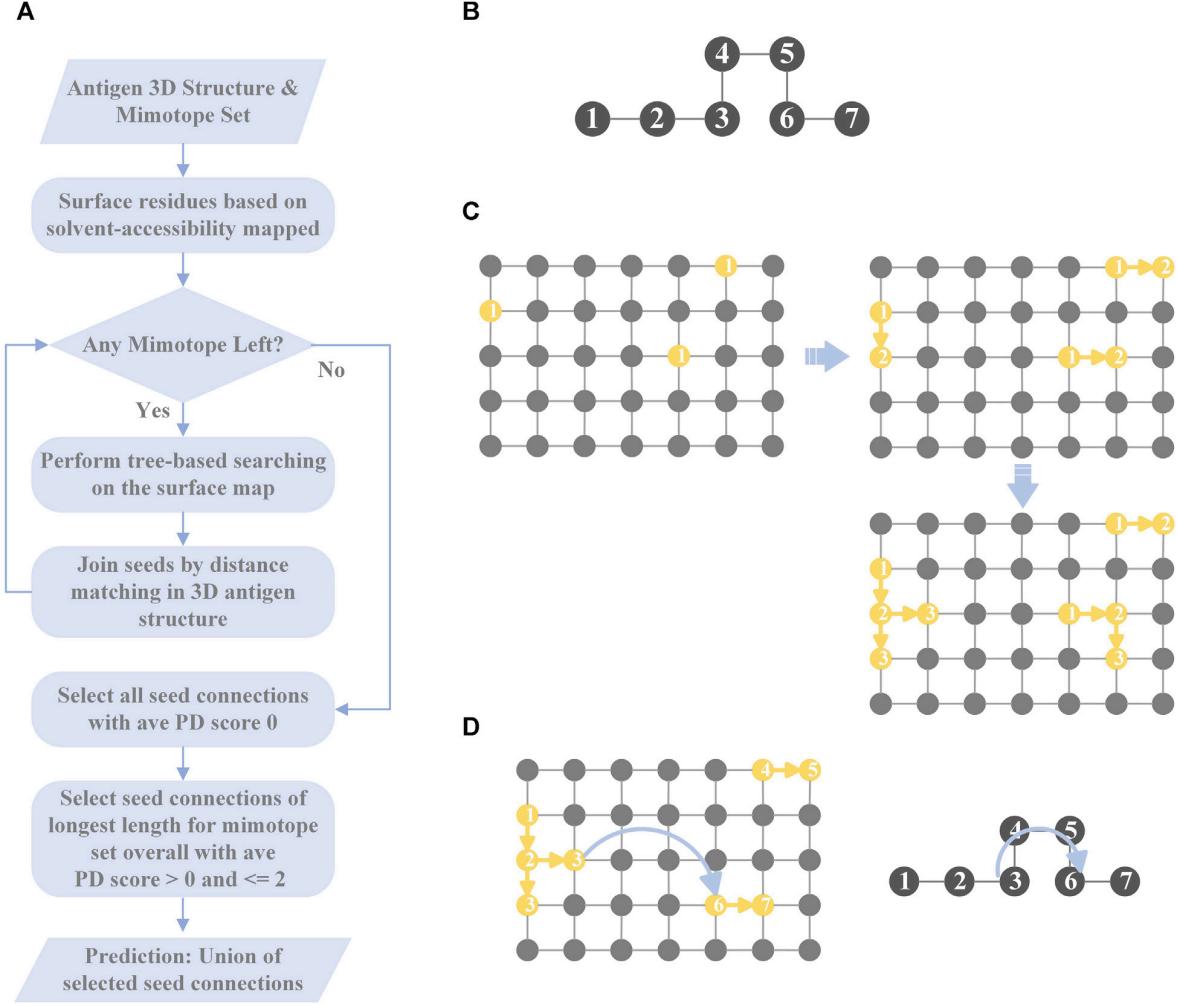
Overview of MimoTree algorithm. A flowchart of MimoTree algorithm is shown in **(A)** and an example of the tree-based search starting from residue 1 of the mimotope sequence given in **(B)** is shown in **(C)**. An example of the seed connection process is shown in **(D)**.

MimoTree is a Depth First Search (DFS)-based algorithm. The inputs to MimoTree are a protein data bank (PDB) file of the structure of the antigen of interest and a mimotope set. MimoTree initially creates a surface map of the input antigen structure. For each individual mimotope in the dataset, MimoTree performs a DFS tree-based search of the mimotope sequence to identify surface segments or seeds on the antigen surface that match to the mimotope. MimoTree then connects seeds on the antigen surface that are within a physically reasonable distance based on the 3D structure of the antigen as described above. The final output prediction for the mimotope set is the union of i) all seed connections (the concatenation of seeds matched to the antigen surface) with average PD scores of 0 (perfect matches) and ii) seed connections of the longest length for the mimotope set overall with average PD score >0 and ≤2. A detailed description of the method is outlined in Figure 1.

#### 2.5.2 Creation of a surface map

For the antigen, MimoTree creates a dictionary where each surface residue is a key, and a list containing the adjacent surface residues of each key is represented as the corresponding value. The maximum alpha-carbon to alpha-carbon distance at which two residues are treated as adjacent to each other is 4 Å.

#### 2.5.3 Tree-based searching to identify seeds

For each mimotope in the set, starting from the first amino acid, MimoTree identifies all of the residues on the protein surface that match. If the property distance between two amino acids is within the pre-defined range (described above), the amino acids are considered as matching in this algorithm. For each surface residue that matches the first residue in the mimotope, MimoTree checks the surface map to see if any adjacent residues match the second residue in the mimotope. If so, it continues searching for the next residue in the mimotope sequence. Otherwise, it terminates that search and starts searching in a different direction from that residue. If that fails, the algorithm starts searching from the next antigen surface residue that matches the first mimotope residue. After finding all possible mimotope matching segments on the antigen surface starting from the first residue of the query mimotope, MimoTree starts a new round of surface searching from the second residue of the mimotope. It continues and completes the surface searching starting from each residue in the mimotope. Finally, the output is all the seeds (matching segments) on the antigen surface that match any part of the query mimotope sequence.

This tree-based searching is based on the DFS algorithm. It starts with a matching surface residue, searches from one direction to the end of that path, and then returns to the previous level to start the search from another direction until the end. Each surface search starting from a residue stops only after all possible paths have been searched, ensuring a comprehensive surface search.

#### 2.5.4 Seed connections

After finding all of the seeds (the residues on the antigen surface that partially match the mimotope), MimoTree tries to join the seeds based on the sequence order of the mimotopes to form “seed connections.” The algorithm connects seeds if the mimotope gap in an extended linear conformation is sufficiently long to extend from the one seed to the other seed. That is, the length of the gap in the mimotope sequence, assuming an extended linear conformation, is compared to the distance in the 3D antigen structure from the last residue of the first seed to the first residue of the next seed. If the maximum length of the mimotope gap is greater than or equal to the distance on the 3D antigen structure between the ends of seeds that are being connected, then this mimotope can be mapped at that position (to that epitope) on the antigen surface. In the hypothetical example of a 7-residue mimotope, where residues 1-3 and 6-7 map to the epitope (Figures 1B, D), the mimotope gap of residues 4-5 is allowed if the length of residues 4-5 plus one residue in an extended linear conformation is greater than or equal to the distance between the residues in the 3D antigen structure that map to mimotope residues 3 and 6. More specifically, the algorithm considers the N-to-N distance between two adjacent residues in the mimotope as 3.5 Å (Bhagavan and Ha, 2015), so in the example above the maximum distance would be 10.5 Å (3 * 3.5 Å/residue). The N-to-N distance between the corresponding residues on the surface of the antigen (those that match to mimotope residues 3 and 6) is then calculated from the coordinates of the residues in the input PDB file and must be less than the linear mimotope gap length (10.5 Å in the example) for a connection to be made. Seeds of the longest length overall are automatically passed on as seed connections as well.

#### 2.5.5 Selection of the seed connections

Since MimoTree searches for possible seed connections on the antigen surface based on the sequence of mimotope, the longer the seed connection is, the greater the portion of the mimotope that is matched and the more likely it is that a true epitope has been identified. The length of a seed connection excludes gaps (so is length 5 for the example above) and can extend to the limit of the length of the mimotope (7 for the example above). MimoTree balances the length of the seed connection with the exactness of the match based on the average PD score for the seed connection as described above in Section 2.5.1 to determine the final prediction for the mimotope set.

#### 2.5.6 Parameter tuning for MimoTree

MimoTree contains several tunable parameters: the cutoff value for the PD (the degree of amino acid similarity), the N-to-N distance for length considered for gaps, and the average PD for seed connection selection. As described above, we calculate the PD between any two amino acids using the values of the physicochemical descriptors of each standard amino acid and the eigenvalues indicating the weights of different descriptors. When the PD between two amino acids is less than a preset cutoff value, MimoTree considers the two amino acids to be identical. Thus, in the process of mapping mimotopes to the antigen surface, if the property distance between a residue in the mimotope and a residue on the antigen surface is less than the cutoff value, the algorithm aligns the mimotope residue to that position on the antigen surface.

If the cutoff value is decreased, the algorithm will match fewer but more similar mimotope residues to antigen residues. As a result, the output will be possible epitopes that are more similar in property space to the aligned mimotopes. Thus, if the input mimotopes are very similar to the true epitope, reducing the cutoff will improve both sensitivity and precision of the algorithm, but if the input mimotopes are not similar enough to the true epitope, lowering the cutoff will reduce both sensitivity and precision. In other words, lowering the cutoff makes the algorithm more dependent on the similarity or identity between the mimotope and the true epitope, which reduces the robustness of the algorithm. Since lowering the threshold would consider fewer residues, it would also shorten the run time of the program. In practice, we found that a cutoff value of 8 yielded the best results across the test set based on the accuracy and run time.

The N-to-N distance was also varied from 3.4 to 3.8 and set to 3.5 Å; a larger distance will allow more matches but may not be physically realistic. The average PD used when selecting the longest seed connection was varied from 2 to 3. All results obtained by MimoTree herein utilize a PD cutoff of 8, an N-to-N distance of 3.5 Å for gaps, and an average PD of 2 for seed connection selection.

### 2.6 Ensemble methods

In this study, we compare MimoTree to two of the other methods that are available online and test several ensemble approaches. The two methods are PepSurf (Mayrose et al., 2007; Negi and Braun, 2009) and EpiSearch (Mayrose et al., 2007; Negi and Braun, 2009) which have complementary search algorithms (described below). More specifically, ensemble approaches combining PepSurf and EpiSearch as well as combining PepSurf and EpiSearch with MimoTree were examined. The PepSurf and EpiSearch calculations were performed with default parameter settings using their servers (http://pepitope.tau.ac.il/index.html and http://curie.utmb.edu/episearch.html, respectively).

#### 2.6.1 Algorithm description of PepSurf

PepSurf first unfolds the antigen surface onto a surface map containing all surface residues, where a pair of neighboring residues in the map is defined as any two residues within a distance of 4 Å. For each individual mimotope, PepSurf looks for all possible simple paths on the surface map that are the same residue length as the given mimotope. With its color-coding technique, PepSurf is able to find every linear path on the surface map without duplication. Afterward, PepSurf calculates the weight of each path by comparing its similarity to the query mimotope based on a modified BLOSUM62 matrix (Henikoff and Henikoff, 1992; Mayrose et al., 2007). The modified substitution score representing the similarity between amino acids i and j, h (i, j), is calculated as follows:

$$h\left(i,j\right)=\frac{q\left(i,j\right)}{p\left(i\right)f\left(j\right)}$$

where q(i, j) is the observed probability of occurrence for each i, j pair in the original BLOSUM62 matrix; p(i) and f(j) are the probabilities of occurrence for i and j in the phage display library and in the original BLOSUM62 matrix, respectively (Mayrose et al., 2007). For each mimotope, the surface path with the highest weight is selected. Finally, PepSurf clusters all the selected surface paths and scores them with respect to their similarity to their corresponding mimotopes to obtain the final prediction results for the mimotope set (Mayrose et al., 2007).

#### 2.6.2 Algorithm description of EpiSearch

EpiSearch first generates surface patches centered on each antigen surface residue using a preset radius value. These overlapping patches can cover the entire antigen surface. The type and number of amino acids contained in each surface patch and in each mimotope sequence are stored in separate matrices. Then, EpiSearch calculates the number of residues in each surface patch that are “identical” to any amino acid in the mimotope, where a pair of residues with a property distance (as defined above) less than or equal to a preset value is defined as a pair of identical residues. For each individual mimotope, EpiSearch scores all surface patches based on the degree of matching of each patch with the mimotope. The highest scoring patch is selected as the predicted patch corresponding to the query mimotope. After obtaining the predicted patches for all the input mimotopes in the set, the individual residues in the predicted patches that match to the corresponding mimotope are predicted to be part of the conformational epitope of the antigen (Negi and Braun, 2009).

#### 2.6.3 Creating an ensemble prediction with PepSurf and EpiSearch

To assess the complementarity of PepSurf and EpiSearch approaches in practice, we examined the performance of taking either the union or the intersection of the top-scoring predictive epitopes from each. First, PepSurf and EpiSearch were used to make predictions for each mimotope set-antigen pair independently using default parameters. For PepSurf, the BLOSUM62 substitution matrix and a gap penalty of −0.5 were used. For EpiSearch, a patch size which of 12 Å (which represents the radius value of the surface patches), a PD cutoff of 10, and an accuracy cutoff of 3 (which indicates the maximum number of mismatches allowed in each surface patch) were used. For EpiSearch, all residues in the top solution were considered. The intersection method takes the intersection of the top-scored solutions from EpiSearch and PepSurf. The union significantly improved the sensitivity of the existing methods while intersection decreased performance, so only the results of the union were analyzed further as described below.

#### 2.6.4 Creating ensemble predictions with MimoTree, PepSurf, and EpiSearch

**FIGURE 2:**
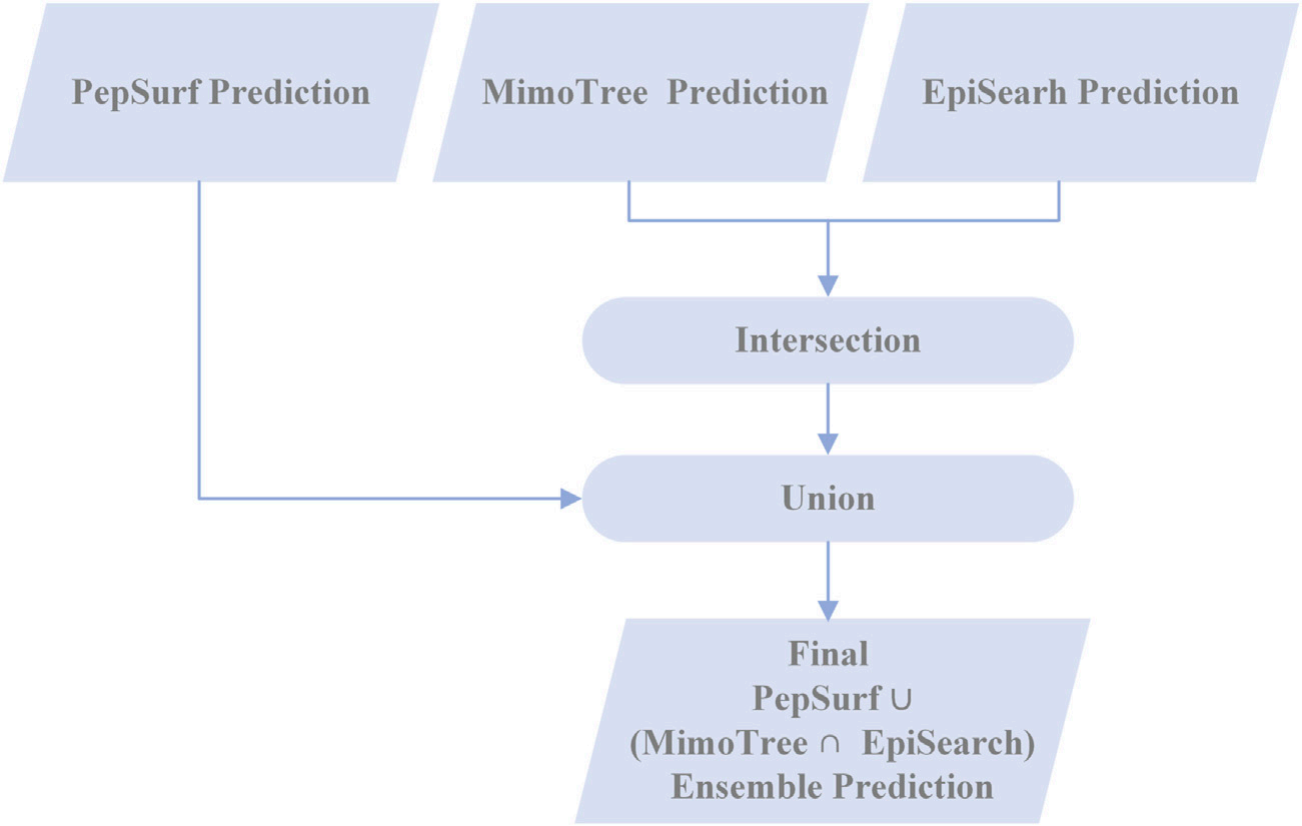
Flowchart of the PepSurf ∪ (MimoTree ∩ EpiSearch) ensemble approach.

In an effort to enhance the performance of MimoTree, two different ensemble approaches for combining the results of MimoTree, PepSurf, and EpiSearch were evaluated. For both, MimoTree, PepSurf and EpiSearch were run individually for each input mimotope set to predict the corresponding epitope on the antigen structure using default parameters as described above. First, MimoTree, PepSurf, and EpiSearch results were combined by majority vote; that is if a residue was in the prediction from at least two of the three methods, it was retained in the final ensemble prediction. A second ensemble approach involved taking the union of the predictions from Pepsurf and the intersection of the results from MimoTree and EpiSearch. PepSearch was chosen as the base prediction in this ensemble because it is the method with the highest average precision over the test set. The goal was to balance improved sensitivity and precision, while limiting the overall size and “density” of the prediction, where the density is the size of the prediction (in terms of number of residues) divided by the size of the antigen. Overall, 95% of all epitopes in the PDB have a solvent-accessible surface area of no more than 2000 Å^2^, and an epitope typically contains no more than 40 amino acids (Mayrose et al., 2007; Huang et al., 2008). See Figure 2 for an overview of the second approach.

### 2.7 Statistical analysis for each solution

To statistically evaluate the performance of the proposed methods, we use sensitivity, precision, Matthews Correlation Coefficient (MCC), and *p*-value on the predictions produced by the various methods.

#### 2.7.1 Sensitivity

Sensitivity is defined as the degree of coverage of the true epitope by the prediction. It is the number of residues correctly predicted divided by the number of residues in the true epitope or:

$$sensitivity=TP/\left(TP+FN\right)$$

where TP is the number of true positives in the prediction and FN is the number of residues in the true epitope that are not in the prediction.

#### 2.7.2 Precision

Precision is the number of correctly predicted residues divided by the number of residues in the prediction. It is calculated as follows:

$$precision=TP/\left(TP+FP\right)$$

where FP is the number of residues in the prediction that are not in the true epitope.

#### 2.7.3 MCC

The Matthews Correlation Coefficient (MCC) is a coefficient of correlation between observed and predicted binary classifications. It is calculated as follows:

$$MCC=\left(TP*TN-FP*FN\right)/\sqrt{\left(TP+FP\right)\left(TP+TN\right)\left(TN+FP\right)\left(TN+FN\right)}$$

where TN is the number of residues in the antigen that are not part of the epitope.

#### 2.7.4 *P*-value

The *p*-value is defined as the probability that a random prediction for a given antigen can perform as well as or better than the prediction obtained by the various methods. The hypergeometric (HG) distribution describes the number of times that n objects of a specified type are successfully drawn (without replacement) from a finite number of N objects (containing M objects of the specified type). The probability of drawing k objects of the specified type is represented as follows:

$$P\left(X=k,n,N,M\right)=\frac{C_{M}^{k}C_{N-M}^{n-k}}{C_{N}^{n}}$$

where k∈{1, 2, … , min (n, M)} and $C_{M}^{k}$ is determined as the number of combinations of M objects taken k at a time (Berkopec, 2007).

In evaluating the performance of the various methods across the test set, the *p*-value calculated based on the HG distribution represents the probability of randomly drawing n residues from an antigen containing N residues, of which M residues are in the prediction, and k or more residues are in the true epitope. A prediction with a *p*-value less than 0.05 is considered to be statistically significant. *p*-values are calculated as follows:

$$P\left(X\geq k,n,N,M\right)=\sum_{X=k}^{\min\;\left(n,M\right)}P\left(X,n,N,M\right)$$

A one-sided Wilcoxon signed-rank tests, a non-parametric statistical hypothesis test, was also performed to test the hypothesis that MimoTree has greater sensitivity or precision than PepSurf or EpiSearch (Conover, 1999).

## 3 Results

### 3.1 MimoTree and comparison to existing methods

MimoTree was first validated on three test cases from the set (1EJ6, 1AVZ, and 1JRH) by giving it a “near exact” match to the epitope as the input “mimotope” (see Supplementary Table S2). Specifically, for a nearly contiguous stretch of the epitope the linear sequence of the antigen was given as the “mimotope”; as such gaps were included in the input mimotope. The resulting sensitivities all agreed approximately with the percentage of the epitope that was given as the input mimotope, indicating that the algorithm was working as expected.

The performance of MimoTree was then assessed over the full test set using the antigen structures from antigen-antibody complexes (Table 3), as well using antigen-alone structures as input (Table 4). As expected, the sensitivity is somewhat diminished when using antigen-alone compared to antigen-antibody complex structures. The average sensitivity over the test set dropped from 0.52 to 0.33 and the average precision from 0.17 to 0.12. None of the other published methods have been evaluated on antigen-alone structures, a more stringent test.

**TABLE 3:** Performance of MimoTree.

PDB ID	Sensitivity	Precision	MCC	*p*-value	Size	Density (%)
1JRH	0.86	0.27	0.32	1.72e-03	45	47.4
1BJ1	0.53	0.26	0.16	6.37e-02^a^	34	36.2
1G9M	0.38	0.05	0.04	1.90e-01^a^	94	30.8
1E6J	0.93	0.14	0.25	1.90e-04	96	45.7
1N8Z	0.44	0.09	0.14	2.86e-03	82	14.1
1IQD	0.44	0.16	0.11	8.64e-02	45	28.9
1YY9	0.30	0.08	0.11	1.56e-02	71	11.6
2ADF	0.44	0.21	0.20	9.16e-03	34	18.0
1AVZ	0.40	0.18	0.06	1.87e-01^a^	34	33.0
1HX1	0.45	0.29	0.18	3.74e-02	31	27.7
Average	0.52	0.17	0.16		56	29.3

^a^ Indicates that the prediction was not statistically significant.

**TABLE 4:** Performance of MimoTree using unbound antigen structures.

PDB ID	Bound PDB ID	Sensitivity	Precision	MCC	*p*-value	Size	Density (%)
1VPF	1BJ1	0.53	0.27	0.14	9.32e-02^a^	37	36.3
3DNO	1G9M	0.23	0.03	−0.04	0.62e-02	96	27.4
1A8O	1E6J	0.86	0.30	0.45	3.79e-03	40	19.1
1D7P	1IQD	0.44	0.13	0.07	1.71e-02	52	32.7
1NQL	1YY9	0.30	0.08	0.10	4.13e-01	76	12.2
1AO3	2ADF	0.00	0.00	−0.13	0.62e-01^a^	31	16.6
1AVV	1AVZ	0.27	0.13	0.02	1.68e-03	30	19.9
1I6Z	1HX1	0.00	0.00	−0.29	0.73e-01^a^	32	23.7
Average		0.33	0.12	0.04		49	23.5

^a^ Indicates that the prediction was not statistically significant.

**FIGURE 3:**
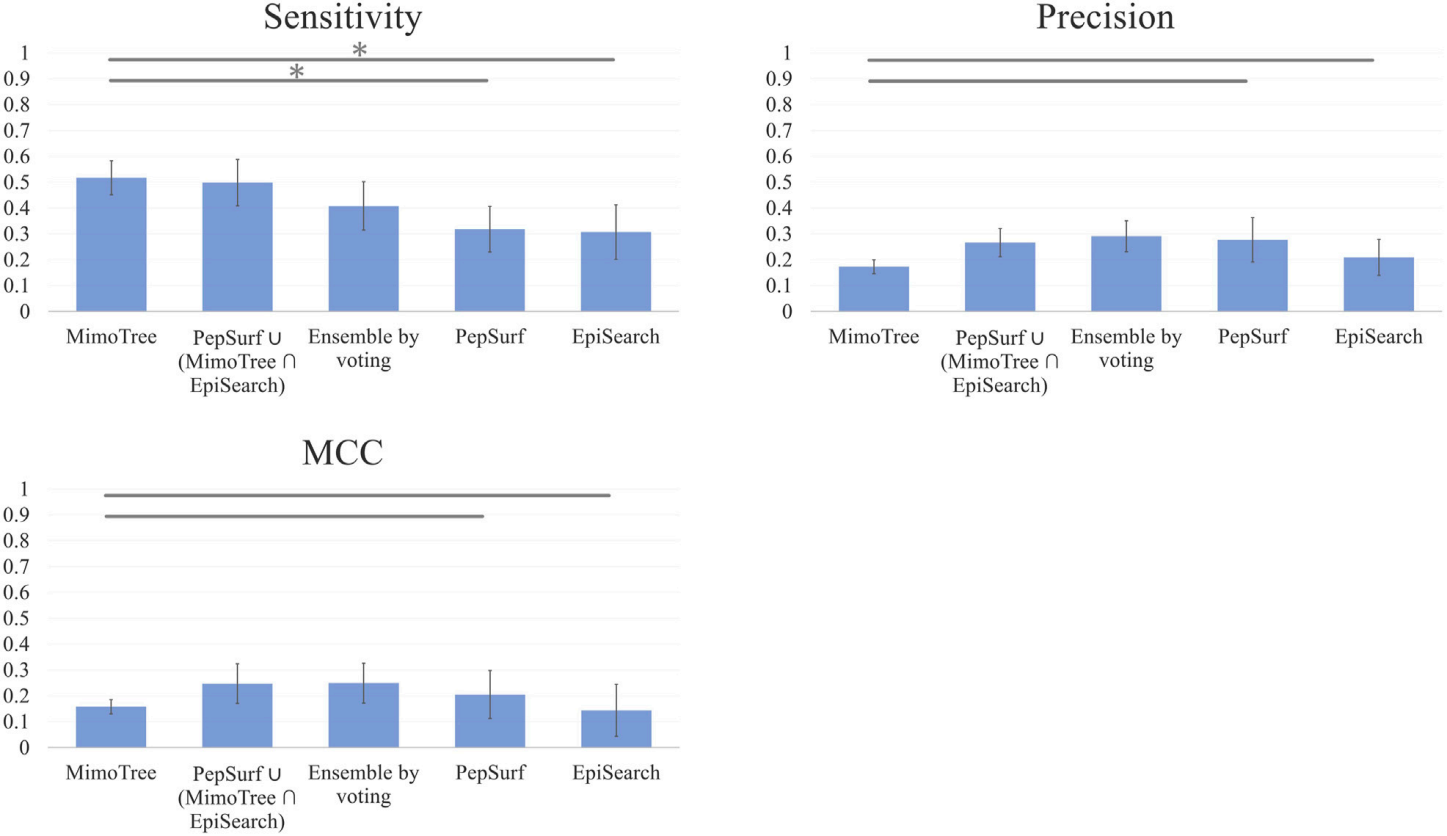
Bar plots of Mean Sensitivity, Precision, and MCC over ten case mimotope-to-epitope test set by approach with Standard Deviation indicated by the error bars. A * indicates difference is significant.

The average sensitivity of MimoTree is improved relative to Pepsurf and EpiSearch over the test set (0.52 vs 0.32 and 0.31, respectively; see Table 5). Statistical significance tests comparing MimoTree to PepSurf and EpiSearch indicate that the sensitivity is significantly improved (*p*-values <0.05) while the precision is largely maintained (Table 6; Figure 3).

**TABLE 5:** Performance of union of PepSurf and EpiSearch compared to PepSurf and EpiSearch.

PDB ID	Union			PepSurf			EpiSearch		
	Sen	Pre	Size/Den (%)	Sen	Pre	Size/Den (%)	Sen	Pre	Size/Den (%)
1JRH	1.00	0.38	44/46.3	0.79	0.44	25/26.3	0.21	0.13	23/24.2
1BJ1	0.11	0.07	28/29.8	0.06	0.06	20/21.5	0.06	0.06	18/19.4
1G9M	0.38	0.14	58/19.0	0.38	0.17	29/9.5	0.00	0.00	29/9.5
1E6J	0.93	0.33	39/18.6	0.00	0.00	18/8.6	0.93	0.62	21/10.0
1N8Z	0.69	0.31	36/6.2	0.44	0.50	14/2.4	0.56	0.31	29/5.0
1IQD	0.63	0.24	42/26.9	0.25	0.15	26/16.7	0.63	0.40	25/16.0
1YY9	0.00	0.00	36/5.9	0.00	0.00	13/2.1	0.00	0.00	23/3.8
2ADF	0.56	0.36	25/13.2	0.56	0.75	12/6.4	0.13	0.13	16/8.5
1AVZ	0.60	0.23	40/38.8	0.60	0.60	15/14.6	0.00	0.00	25/24.3
1HX1	0.65	0.31	42/37.5	0.10	0.10	21/18.8	0.55	0.44	25/22.3
Avg	0.56	0.24	39/24.2	0.32	0.28	19/12.7	0.31	0.21	23/14.3

Sen = Sensitivity, Pre = Precision, Den = Density.

**TABLE 6:** *p*-values for comparisons between MimoTree and existing methods.

	MimoTree vs PepSurf	MimoTree vs EpiSearch
Sensitivity	0.0371	0.0273
Precision	0.7842^a^	0.6875^a^
MCC	0.7217^a^	0.4609^a^

^a^ Difference between the two groups of data is not statistically significant.

### 3.2 Ensemble approaches

The union of PepSurf and EpiSearch also improved the average sensitivity (0.56 vs 0.32 and 0.31, see Table 5) while maintaining the average precision across the test set relative to the individual methods alone. This result indicates that in fact the two methods are complementary. However, the average size of the prediction and density (number of residues in the prediction/number of residues in the antigen) increased as well.

Taking the ensemble of MimoTree, PepSurf, and EpiSearch by majority voting gives the best average precision overall (0.29) and a low prediction size and density (20 and 13.5%). The average sensitivity, while still higher than that for PepSurf or EpiSearch, is only 0.41. Next, we chose PepSurf which has relatively high precision (0.28) and low prediction size/density (19/12.7%), as the base method to which we added the intersection of the prediction from MimoTree and EpiSearch. This yielded an average sensitivity of 0.50 (similar to MimoTree) but with a higher precision (0.27 vs 0.17) and a much smaller prediction size/density (29/18.9%) (Table 7).

**TABLE 7:** The performance of PepSurf ∪ (MimoTree ∩ EpiSearch) ensemble across the test set.

PDB ID	Sensitivity	Precision	MCC	*p*-value	Size	Density (%)
1JRH	0.86	0.34	0.42	7.23e-05	35	36.8
1BJ1	0.06	0.08	−0.17	1.49e-01^a^	25	26.6
1G9M	0.38	0.12	0.15	1.94e-02	43	14.1
1E6J	0.93	0.34	0.52	3.90e-10	38	18.1
1N8Z	0.63	0.38	0.47	3.21e-11	26	4.5
1IQD	0.56	0.25	0.27	2.11e-03	36	23.1
1YY9	0.00	0.00	−0.03	5.64e-01^a^	17	2.8
2ADF	0.56	0.60	0.54	6.44e-08	15	7.9
1AVZ	0.60	0.32	0.22	3.41e-03	28	27.2
1HX1	0.35	0.23	0.08	1.51e-01^a^	31	27.7
Average	0.50	0.27	0.25		29	18.9

^a^ Indicates that the prediction was not statistically significant.

**FIGURE 4:**
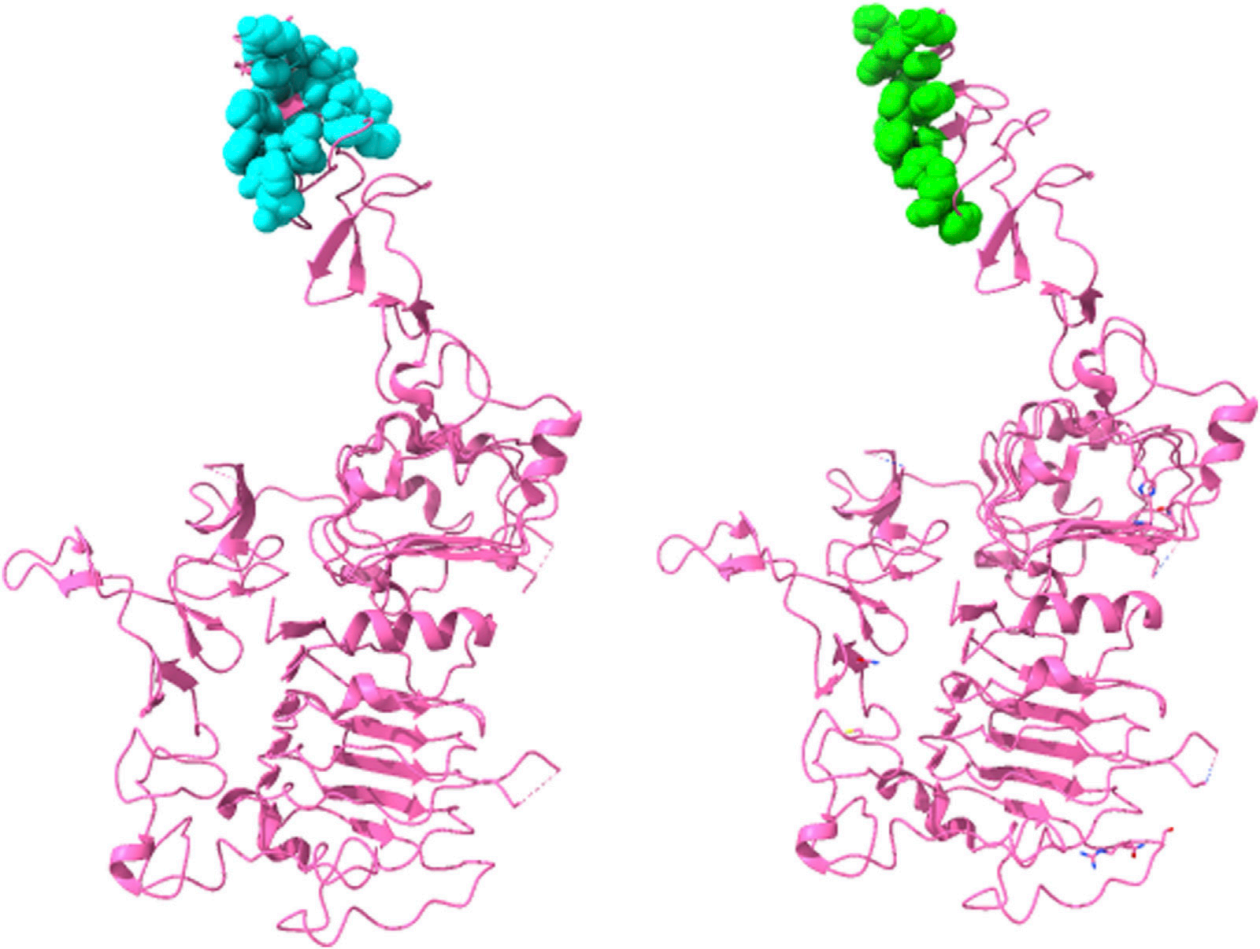
Structure of Herceptin Fab-Her-2 complex (1N8Z) showing the epitope predicted by the ensemble approach *versus* the true epitope. The antigen is shown in pink ribbon with residues in the prediction on the left in cyan in CPK spheres and the true epitope on the right in green.

**FIGURE 5:**
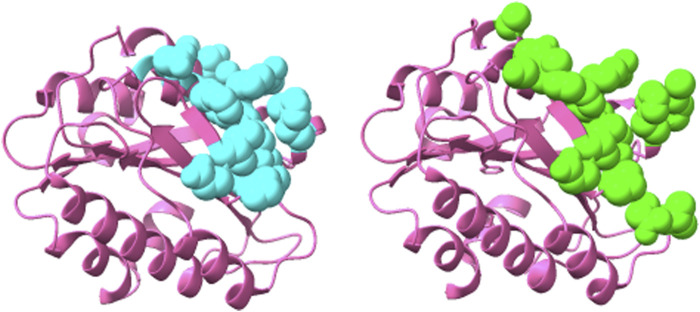
Structure of 82D6A3 IgG-Von Willebrand factor (2ADF) showing the epitope predicted by the ensemble approach *versus* the true epitope. The antigen is shown in pink ribbon with the prediction on the left in cyan in CPK spheres and the true epitope on the right in green.

Thus, this ensemble approach, PepSurf ∪ (MimoTree ∩ EpiSearch), yielded the best set of performance metrics overall (see Table 8). Images showing the prediction vs the true epitope on the antigen structure are given in Figures 4, 5 for 1N8Z and 2ADF and in the Supplementary Material for all other test cases (Supplementary Figures S1-S8). These figures serve as a guide for what the statistics reflect in terms of the mapping of the mimotope onto the antigen structure and have not generally been provided for other published methods.

**TABLE 8:** Average performance metrics by approach.

	Average sensitivity	Average precision	Average size	Average density (%)
MimoTree	0.52	0.17	56	29.3
PepSurf	0.32	0.28	19	12.7
EpiSearch	0.31	0.21	23	14.3
Union of PepSurf + EpiSearch	0.56	0.24	39	24.2
Ensemble by voting^a^	0.41	0.29	20	13.5
PepSurf ∪ (MimoTree ∩ EpiSearch)	0.50	0.27	29	18.9

^a^ Ensemble of MimoTree, PepSurf, and EpiSearch.

## 4 Discussion

In this study, we developed the novel method MimoTree as well as an ensemble approach combining MimoTree with PepSurf and EpiSearch for predicting the epitope on the antigen surface from mimotope data. MimoTree and the PepSurf ∪ (MimoTree ∩ EpiSearch) ensemble approach outperform PepSurf and EpiSearch by improving sensitivity while maintaining precision.

By examining the union of PepSurf and EpiSearch, we showed that PepSurf and EpiSearch use different but complementary algorithms for the process of mapping mimotopes to an antigen structure that include differences in analyzing, scoring, and clustering the locations. EpiSearch first divides the surface of the antigen into overlapping surface patches. These patches are circular regions centered on each of the surface residues. EpiSearch then calculates the similarity between the surface residues contained in these circular patches and each input mimotope and obtains an initial epitope prediction. The manner by which EpiSearch divides the antigen surfaces tends to make this algorithm more suitable for predicting compact epitopes; if the true epitope is loosely distributed in multiple patches, EpiSearch is less likely to predict it correctly as observed in the test set (e.g., for 1G9M, 1YY9, and 2ADF). Meanwhile, PepSurf uses color-coding techniques to find all possible linear paths on the antigen surface. These linear paths of different shapes may be able to traverse the entire antigen in one direction. Thus, these two methods are somewhat complementary in their surface searching approach. EpiSearch accurately locates specific residues in the epitope when it finds the right patches, but it also sometimes locates the wrong patches and therefore misses the correct epitope entirely. Conversely, PepSurf is better at finding the correct approximate epitope location but is less likely to find the specific residues within it.

MimoTree algorithm is novel in that it addresses the possibility of gaps in mimotope-epitope mapping and it considers the 3D structure of the antigen in its final epitope connection step. If a mimotope cannot be continuously mapped on the antigen surface, several existing methods, including PepSurf, reflect this feature in the scoring of this path, i.e., a gap penalty with a negative value is added to the score. However, gaps in the mimotope sequences matched to the epitope (as well as in the epitope itself if it is conformational) are often seen since mimotopes mimic the structure and sequence of epitopes, but are usually not identical to the epitope. To solve this problem, MimoTree first unfolds the antigen surface into a surface map. For each input mimotope, MimoTree looks for each of its sequence fragments (seeds) on the antigen surface and tries to join these seeds in the sequence order of the original mimotope with a distance restraint based on the 3D structure of the antigen and the maximal length of the mimotope (if in an extended linear conformation). If the distance between the ends of the two seeds on the 3D antigen structure is less than or equal to the distance between corresponding residues in the mimotope (assuming a linear extended conformation), then the seeds are connected. MimoTree predicts the mimotope to map to that position on the antigen surface.

Because MimoTree uses a depth-first search method during the searching process, the longer the length of the seed connection (matching the mimotope to the antigen surface), the greater the degree of match overall. Gaps in the seed connections of longest length that were incorporated into the MimoTree predictions for each case in the test set were analyzed. In 3/10 test cases, there were two gaps each in the longest seed connections, while in 4/10 cases there was one gap in the seed connections. The average gap length was 3.1 residues and the most common amino acids found in the gaps were Arg, Lys, Pro, Leu, Asn, and Glu. In future work these trends could be analyzed over a larger sample size.

While evaluating the performance of the various methods, we found that EpiSearch and PepSurf were unable to accurately predict any of the residues in the epitope of the 1YY9 antigen (epidermal growth factor receptor (EGFR)). MimoTree does find some matches between the mimotopes and the epitope for this test case, however, the prediction is loosely dispersed over the two main domains of the structure (Domain III, which interacts with the antibody, and Domain I, which would interact with EGF), and has low sensitivity and precision (0.30 and 0.08). This is a particularly difficult test case, in that the residues in the epitope are loosely distributed in that the true epitope consists of 20 residues but the longest contiguous stretch is of four residues, which greatly increases the difficulty of algorithmically predicting the location of the epitope. 1BJ1 is also a difficult case in that the antigen (vascular endothelial growth factor (VEGF)) is secreted as a dimer of two identical monomers and in the structure an antibody is bound at each of the identical dimer poles. MimoTree does locate both poles on the monomer of the dimer but the prediction size and density are relatively high (see Supplementary Figure S9).

For both MimoTree and the PepSurf ∪ (MimoTree ∩ EpiSearch) ensemble approach the sensitivity is highest for 1EJ6 (0.93), but with MimoTree the precision is relatively low (0.14). With the ensemble approach the sensitivity remains high and the precision increases. The true epitope is identified with high precision but a second patch on the antigen is also highlighted (Supplementary Figure S4). Two of the best cases based on the ensemble approach are 1N8Z and 2ADF both of which balance sensitivity and precision and have relatively low densities (see Figures 4, 5).

In general, our results indicate that sensitivity, precision, and density must all be considered when evaluating a given prediction and a mimotope mapping approach overall. In particular, predictions that balance sensitivity and precision tend to map most closely to the true epitope. The results also suggest that ensemble approaches likely are more effective at achieving that balance assuming that the methods included employ complementary strategies. The current limitations of MimoTree and the PepSurf ∪ (MimoTree ∩ EpiSearch) ensemble approach, however, still lie in the relatively high false positive rate. In future work, adding a machine learning component to the MimoTree combination step and/or the ensemble formation may improve the results further by reducing false positives. Mimotope methods like MimoTree and the PepSurf ∪ (MimoTree ∩ EpiSearch) ensemble approach will further our understanding of the characteristics of antigen-antibody binding interactions, may for certain pathogens lead to the design of improved vaccine with limited potential for resistance, and can aid in the rapid design of more specific and sensitive diagnostic immunoassays.

## Data Availability

The original contributions presented in the study are included in the article/Supplementary Material, further inquiries can be directed to the corresponding author.
