# Supplementary material for: Computational identification of antibody-binding epitopes from mimotope datasets
Source: Front Bioinform. 2024 Feb 23;4:1295972. doi: 10.3389/fbinf.2024.1295972 (PMC10920257; doi:10.3389/fbinf.2024.1295972)
Supplement: Supplementary file 1 [file DataSheet1.docx]

Supplementary Materials

COMPUTATIONAL IDENTIFICATION OF ANTIBODY-BINDING EPITOPES FROM MIMOTOPE DATASETS

Rang Li^1^, Sabrina Wilderotter^1^, Madison Stoddard^2^, Debra Van Egeren^3^, Arijit Chakravarty^2^, Diane Joseph-McCarthy^1^*

^1^Department of Biomedical Engineering, Boston University, Boston, MA, USA

^2^Fractal Therapeutics Inc., Cambridge, MA, USA

^3^Stanford Cancer Institute, Stanford University School of Medicine, Stanford, CA, USA

*** Correspondence:**Diane Joseph-McCarthy
djosephm@bu.edu

Keywords: conformational epitope, mimotopes, structure-based mimotope mapping, antigen-antibody binding site prediction, immunodiagnostics design

**Table S1. Mimotope set and corresponding protein structure used as the training set**

| PDB identifier | Mimotope set |
| --- | --- |
|  |  |
| 1JRH | EHKWY  GVKSY  ENKWY  EAKSY  EEKRH  QAKSY  AVKEY  QAKEY  EAKNY  EGKWY  EVKSY  ETKVY  EEKSY  EHKVY  EQKFY  EMKTY  KVKNY  MNKWH  GVKIH  GLKTY  SVKSY  IAKSY  LVKTY  YGVKN  YGTKN  YGYKN  HGVKN  YGHKN  YGLKN  HGLKN  NSPWR  NGGWR  NSPWL  NTPWS  NAPWT  NGPWK  NPRWQ  NSPWV  NSPWA  NSPWS  NSPWF  NTPWV  NAPPN  NAPWE  NGPWV  NGPWR  NSVFH  NPSWS  NPTWT  NPTWQ  NPTYR  NPAYR  NPGWV  NPRWM  NGLWT  NGDYE  NGSWR  NGYYE  NGTWR |
| 1BJ1 | YDFTHY  YEFQHY  YEFTHY  YDFGHY  YDFSHY  YEFSHY  TWTGE  TWTGT  TWTGQ  NTWT  NTWD  YYRNAS  YYTTRS  YYEGSS  YYRQRG  YYTGRS  YYTNTS  YYRKGS  YYTGSS  YYRSGS  YYTNRS  YYRNSS  YYKESS  YYRDAS  YYRQKS  YYKGGS  YYYGAS  YYRGES  YYRSTS  YYRNTS  YYYGTS  YYRGTS  YYINKS  YYYNQS  YYIAKS  YYRDNS  YYWGTS  YYRQNS  YYRQSS  YYKNTS  YYIERS |
| 1G9M | CLHIRVNETAYRVC  CEFFQQHMLRVPRC  CNMKLKLREMTQRC  CMTRPTSLTQLTGC  CMVRPSNWDALTRC  CDFLREHGMKNPRC  CRSRPTNMTTLRDC  CAAYNATRGTVSAC  CQLLHTWEDKMRKC  CRNGELWLRRPGLC  CSGLRNETFLRC |
| 1E6J | CAHFPPRSQMIADC  CAHFAPGTAMYSDC  CRQFPHSSSMYTDC  CRESRAALERGWWC  CEARTHNEARRRRC  CAAARSTGETSAHY  CYYRMGANYTVGEC  CSVSPLYAYDDPLC  CTQMHEMDPNFPPC  CVTALGPNYTGQEC  CYVQQPWWVLEREC  CADVMGPLVTAAEC  CADVMGPLVTAGEC  CVVFLDVSEAFRDC  VWRCNWF  AASWNGR |
| 1N8Z | CQMWAPQWGPDC  CKLYWADGELTC  CKLYWADGEFTC  CVDYHYEGTITC  CVDYHYEGAITC |
| 1IQD | CMKWSNRSSRWC  QCSKWVNRSRCA  CSKWHNRSKRHC  CSKWANRLVSIC  NCGKWTNRRTCL  QCSRWSNRTSCT  KCSRWTNRHLCD  KCTRWTNRHLCS  KCTRWTNRAHCP  ECTRWSNRSRCF  CGRWFNRSDLHC  QCGRWSNRSYCS  KCGRWSNRSSCT  TCHRWGNRTSCQ  QCHRWANRISCS  QCHTWSNRRSCL  ACTTWSNRSKCP  RCTQWTNRAYCP  ACTQWSNRHMCG  SCHAWSNRRTCR  RCHAWSNRKSCV  ACHEWSNRSTCT  KCGPWSNRSSCT  TCHPFSNRSTCT  KCEPDDPWPQCI  ACKRNHRWGACV  ECGSHAWGRRCK |
| 1YY9 | QFDLSTRRLK  QYNLSSRALK  VWQRWQKSYV  MWDRFSRWYK |
| 2ADF | GDCFFGFLNSPWRVC  RSSYWVYSPWRFISR  MTSPWR  RTSPWR  YRSPWR |
| 1AVZ | IQHRLLPPIP  RLLKPLPPIP  QSRRSLPPIP  AKRAPLPPIP  TGGRPLPPIP  HHIRPLPPIP  SYPRPLPPIP  HSTRALPPIP  RSLRPLPPLPFD  RSLRPLPPPPFP  RSLRPLPPLPVP  RSLRPLPPLPWT  RSLRPLPPLPPL  RSLRPLPPIPGR  RSLRPLPPIPPP  RSLRPLPPLPAP  RSLRPLPPIPLG  RSLRPLPPIPDS |
| 1HX1 | SIEVLRGAMHVAPRR  RRGDHDHDIFHWWVH  GGWYDRKHRRPAPLS  KFFRKKSHYHSRTTS  FLDERNYIKKKRHKL  KKKRHDVEIPHVRAS  MDDRGLLLVKKKKHG  FDGVRKKSRGKSLEY  TELDIYKVWISRDGV |

**Table S2. “Near exact” match test set**

| **1E6J** | | | | **1AVZ** | | | | **1JRH** | | | |
| --- | --- | --- | --- | --- | --- | --- | --- | --- | --- | --- | --- |
| **True Epitope** | **Input Epitope** | **Input Res.** | **Sens,**  **Prec^a^** | **True Epitope** | **Input Epitope** | **Input Res.** | **Sens.,**  **Prec.** | **True Epitope** | **Input Epitope** | **Input Res.** | **Sens.,**  **Prec.** |
| 187 | 201 | I | 0.86,  0.26 | 71 | 71 | T | 0.46,  0.58 | 47 | 47 | K | 0.71,  0.48 |
| 201 | 202 | L |  | 72 | 72 | P |  | 49 | 48 | N |  |
| 203 | 203 | K |  | 73 | 73 | Q |  | 50 | 49 | Y |  |
| 204 | 204 | A |  | 74 | 74 | V |  | 51 | 50 | G |  |
| 205 | 205 | L |  | 75 | 75 | P |  | 52 | 51 | V |  |
| 206 | 206 | G |  | 77 | 76 | L |  | 53 | 52 | K |  |
| 207 | 207 | P |  | 82 | 77 | R |  | 54 | 53 | N |  |
| 208 | 208 | A |  | 86 |  |  |  | 55 | 54 | S |  |
| 209 | 209 | A |  | 90 |  |  |  | 56 | 55 | E |  |
| 210 | 210 | T |  | 117 |  |  |  | 76 | 56 | W |  |
| 212 | 211 | L |  | 118 |  |  |  | 78 |  |  |  |
| 213 | 212 | E |  | 120 |  |  |  | 82 |  |  |  |
| 216 | 213 | E |  |  |  |  |  | 84 |  |  |  |
| 217 | 214 | M |  |  |  |  |  | 98 |  |  |  |
|  | 215 | M |  |  |  |  |  |  |  |  |  |
|  | 216 | T |  |  |  |  |  |  |  |  |  |
|  | 217 | A |  |  |  |  |  |  |  |  |  |

^a^ Sensitivity, Precision


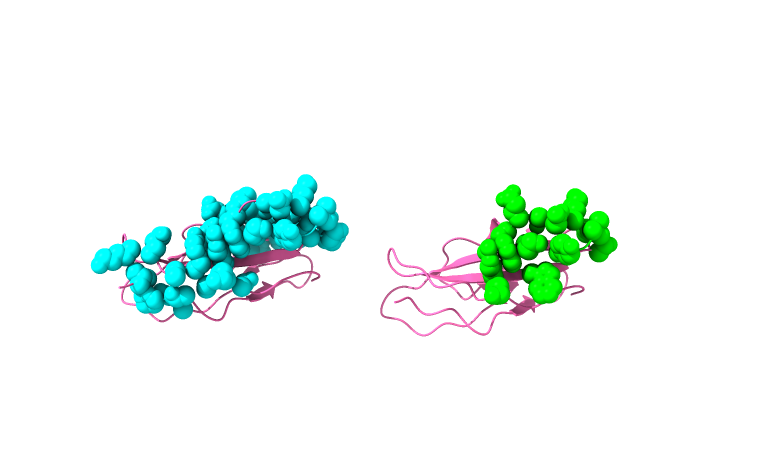


Figure S1. Structure of mAb A6 – IFNgammaR complex (11JRH) showing the epitope predicted by the ensemble approach versus the true epitope. The antigen is shown in pink ribbon with residues in the prediction on the left in cyan in CPK spheres and the true epitope on the right in green.


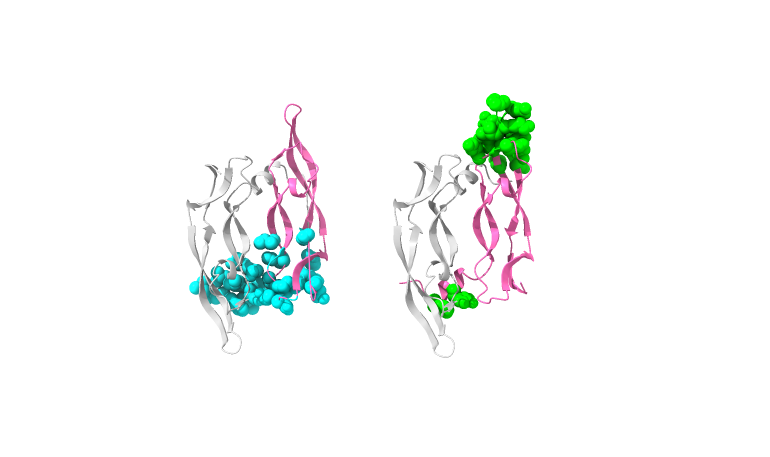


Figure S2. Structure of rhuMAb – Vascular endothelial growth factor complex (1BJ1) showing the epitope predicted by the ensemble approach versus the true epitope. One monomer of the antigen is shown in pink ribbon and the other in grey with residues in the prediction on the left in cyan in CPK spheres and the true epitope on the right in green.


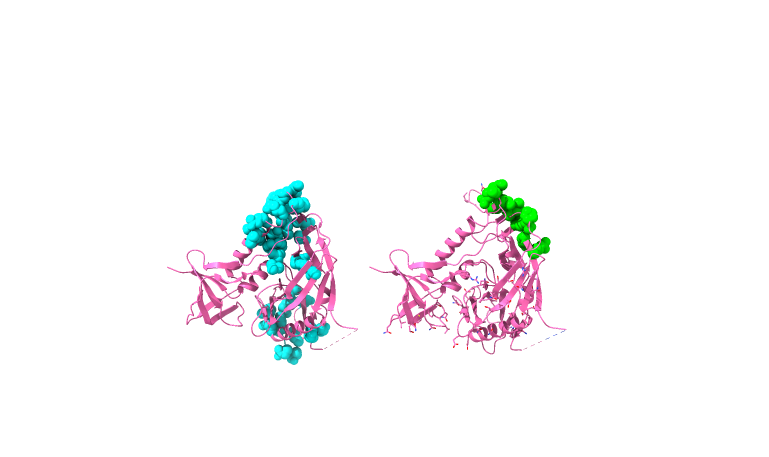


Figure S3. Structure of mAb 17b – Gp120 complex (1G9M) showing the epitope predicted by the ensemble approach versus the true epitope. The antigen is shown in pink ribbon with residues in the prediction on the left in cyan in CPK spheres and the true epitope on the right in green.


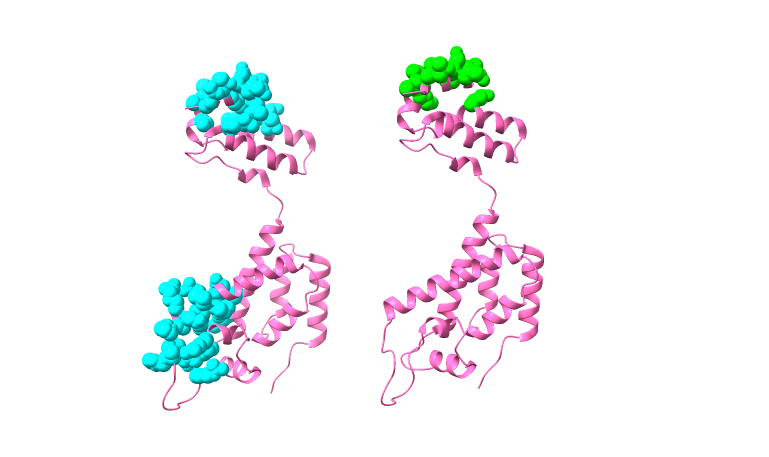


Figure S4. Structure of mAb 13b5 – P24 complex (1E6J) showing the epitope predicted by the ensemble approach versus the true epitope. The antigen is shown in pink ribbon with residues in the prediction on the left in cyan in CPK spheres and the true epitope on the right in green.


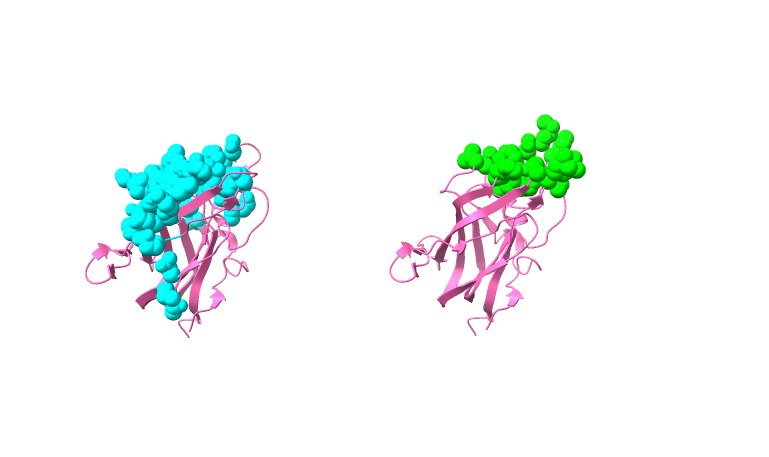


Figure S5. Structure of mAb Bo2C11 – Coagulation factor VIII complex (1IQD) showing the epitope predicted by the ensemble approach versus the true epitope. The antigen is shown in pink ribbon with residues in the prediction on the left in cyan in CPK spheres and the true epitope on the right in green.


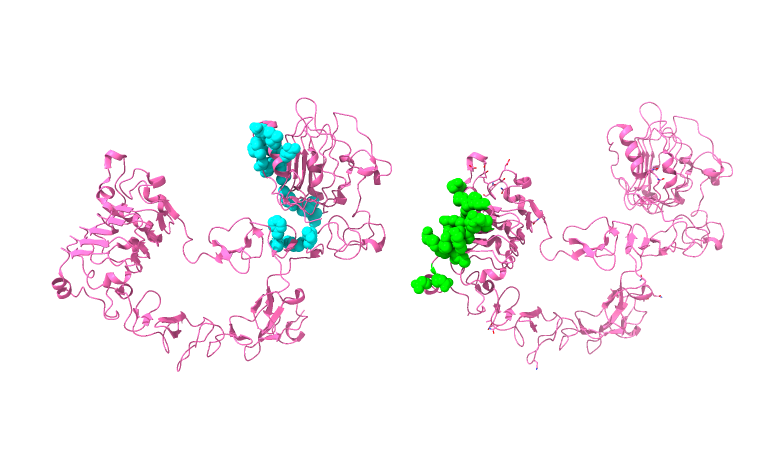


Figure S6. Structure of Cetuximab Fab – Epidermal growth factor receptor complex (1YY9) showing the epitope predicted by the ensemble approach versus the true epitope. The antigen is shown in pink ribbon with residues in the prediction on the left in cyan in CPK spheres and the true epitope on the right in green.


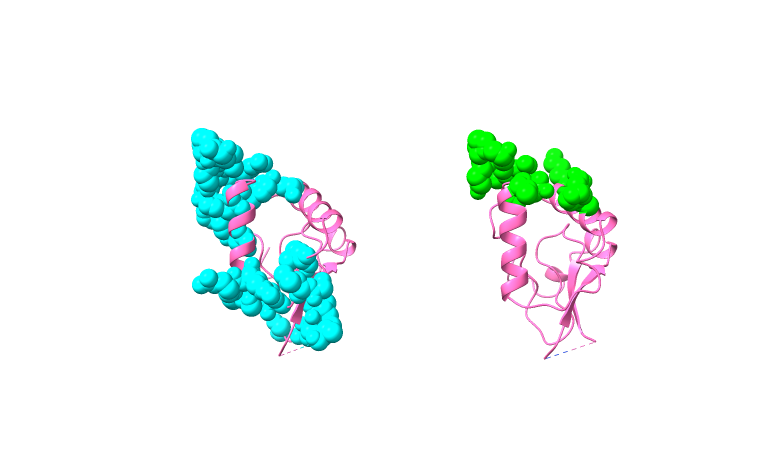


Figure S7. Structure of Fyn SH3 domain – Nef complex (1AVZ) showing the epitope predicted by the ensemble approach versus the true epitope. The antigen is shown in pink ribbon with residues in the prediction on the left in cyan in CPK spheres and the true epitope on the right in green.


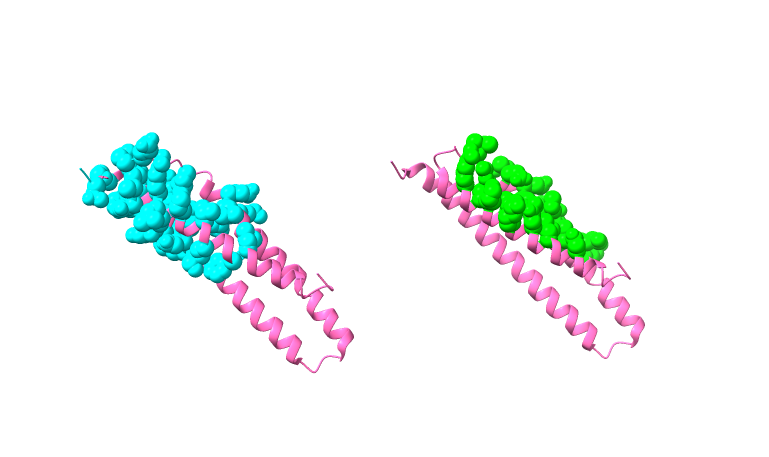


Figure S8. Structure of Bovine Hsc70 – Bag chaperone regulator showing the epitope predicted by the ensemble approach versus the true epitope. The antigen is shown in pink ribbon with residues in the prediction on the left in cyan in CPK spheres and the true epitope on the right in green.


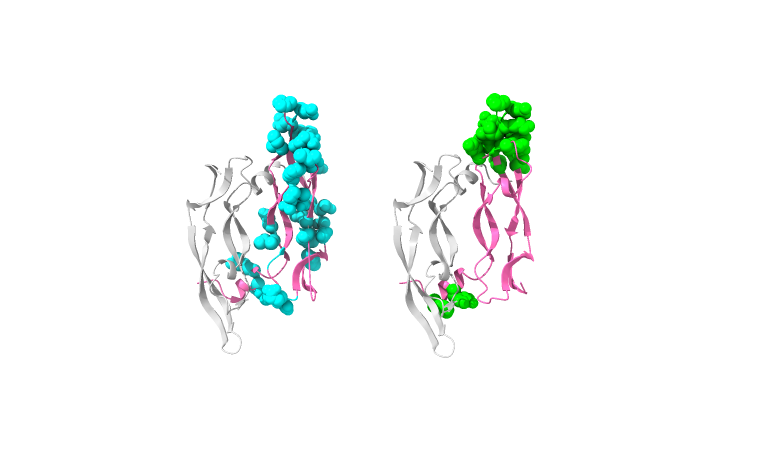


Figure S9. Structure of rhuMAb – Vascular endothelial growth factor complex (1BJ1) showing the epitope predicted by MimoTree versus the true epitope. One monomer of the antigen is shown in pink ribbon and the other in grey with residues in the prediction on the left in cyan in CPK spheres and the true epitope on the right in green.
